# Computational insights into the inhibitory mechanism of type 2 diabetes mellitus by bioactive components of *Oryza sativa L. indica* (black rice)

**DOI:** 10.3389/fphar.2024.1457383

**Published:** 2024-09-23

**Authors:** Kashaf Rasool, Attya Bhatti, Abid Majeed Satti, Rehan Zafar Paracha, Peter John

**Affiliations:** ^1^ Department of Atta-ur-Rahman School of Applied Biosciences (ASAB), National University of Science and Technology (NUST), Islamabad, Pakistan; ^2^ Crop Science Institute (CSI), PARC-National Agriculture Research Center (NARC) Islamabad, Islamabad, Pakistan; ^3^ School of interdisciplinary Engineering and Sciences (SINES), National University of Science and Technology (NUST), Islamabad, Pakistan

**Keywords:** diabetes mellitus, type 2, Oryza, network pharmacology, molecular dynamics simulations, insulin, drug discovery

## Abstract

**Background:**

Type 2 diabetes mellitus is a metabolic disease categorized by hyperglycemia, resistance to insulin, and ß-cell dysfunction. Around the globe, approximately 422 million people have diabetes, out of which 1.5 million die annually. In spite of innovative advancements in the treatment of diabetes, no biological drug has been known to successfully cure and avert its progression. Thereupon, natural drugs derived from plants are emerging as a novel therapeutic strategy to combat diseases like diabetes.

**Objective:**

The current study aims to investigate the antidiabetic potential of natural compounds of *Oryza sativa L. indica* (black rice) in disease treatment.

**Methods:**

Antioxidant activity and alpha amylase assays were performed to evaluate the therapeutic potential of the extract of *Oryza sativa L. indica*. Gas chromatography–mass spectrometry (GC–MS) was used for identification of constituents from the ethanol extract. ADMET profiling (absorption, distribution, metabolism, excretion, and toxicity), network pharmacology, and molecular dynamics simulation were employed in order to uncover the active ingredients and their therapeutic targets in *O. sativa L. indica* against type 2 diabetes mellitus.

**Results:**

GC–MS of the plant extract provided a list of 184 compounds. Lipinski filter and toxicity parameters screened out 18 compounds. The topological parameters of the protein–protein interaction (PPI) were used to shortlist the nine key proteins (STAT3, HSP90AA1, AKT1, SRC, ESR1, MAPK1, NFKB1, EP300, and CREBBP) in the type 2 diabetes mellitus pathways. Later, molecular docking analysis and simulations showed that C14 (1H-purine-8-propanoic acid, .alpha.-amino-2, 3, 6, 7-tetrahydro-1,3,7-trimethyl-2,6-dioxo-) and C18 (cyclohexane-carboxamide, N-furfuryl) bind with AKT1 and ESR1 with a binding energy of 8.1, 6.9, 7.3, and 7.2 kcal/mol, respectively. RMSD (root-mean-square deviation) and RMSF (root-mean-square fluctuation) values for AKT1 and ESR1 have shown very little fluctuation, indicating that proteins were stabilized after ligand docking.

**Conclusion:**

This study suggests therapeutic drug candidates against AKT1 and ESR1 to treat type 2 diabetes mellitus. However, further wet-lab analysis is required to discover the best remedy for type 2 diabetes mellitus.

## 1 Introduction

Type 2 diabetes mellitus (T2DM) is the most common metabolic disorder (Galicia-Garcia et al., 2020), affecting approximately 2.8% of the people worldwide, and it is anticipated to affect more than 11.3% of the population by the year 2026 (Hossain et al., 2024). T2DM is a multifactorial disease that is categorized by hyperglycemia and insulin resistance. Cells in muscle, liver, and fats develop resistance to insulin, resulting in a high level of glucose in blood (hyperglycemia), while cells become deprived of sugar and trigger ß-cell hyper function in order to increase the secretion of insulin (hyperinsulinemia) (Aedh et al., 2023). The higher levels of insulin cannot overcome the reduction in insulin sensitivity; therefore, ß-cell function begins to fail, and the pancreas is unable to keep blood sugar levels in the normal range. Long-term hyperglycemia results in diabetic complications, which includes structural and functional abnormalities of organ systems beginning from the pancreas (Banday et al., 2021).

The phosphatidylinositol 3-kinase (PI3K)/protein kinase B (AKT) signaling pathway is impaired in insulin resistance, and this pathway “is very important for glucose homeostasis, lipid metabolism, and cell proliferation.” (Ramasubbu et al., 2023) AKT serine/threonine kinase 1 (AKT1) carries out translocation of glucose transporter 4 (GLUT4) to the membrane of cells. GLUT4 is the receptor for glucose uptake in body cells. AKT1 also increases glycogen synthesis by inactivating glycogen synthase kinase 3 (GSK3) and affects lipid metabolism by promoting fatty acid synthesis (Huang et al., 2018).

Estrogen receptor 1 (ESR1) plays a crucial role in regulating glucose homeostasis and insulin sensitivity. ESR1 enhances insulin signaling by upregulating the insulin receptor substrate and increases the expression and translocation of glucose transporters. Thus, it helps in glucose uptake in muscle and adipose tissues and lowers the blood glucose level. ESR1 regulates lipid metabolism, and its activation through the drug can improve metabolic health, making it a therapeutic target against the treatment T2DM (Ereqat et al., 2019; Yang et al., 2018).

The T2DM medication course includes oral hypoglycemic agents such as dipeptidyl peptidase-4 (DPP4), and they work by inhibiting the DPP-4 enzyme, which increases insulin secretion, but they have side effects such as pancreatitis. Insulin sensitizers increase insulin sensitivity in cells, but they are associated with weight gain, edema, and risk of heart failure. Secretagogues work by the rapid release of insulin, but they can cause weight gain. Glucagon-like peptide 1 (GLP-1) agonists promote insulin release in response to glucose presence, but they can cause gastrointestinal problems and weight loss. Sodium–glucose co-transporter (SGLT) inhibitors reduce glucose absorption in the kidney and increase the excretion of glucose in urine, but they have the risk of causing diabetic ketoacidosis (Longo et al., 2019; Hu S. et al., 2023). These medications have been known to cause many side effects; therefore, scientists continue to search for novel, natural, safer, and targeted agents against T2DM. Small-molecular inhibitors from natural sources are considered an effective way for targeted treatment of many diseases. Treatment of various diseases with small bioactive constituents from plants in various formulations is often encouraged. According to the World Health Organization (WHO), small bioactive components extracted from plant sources are still used by 80% of the population worldwide. *Oryza sativa L. indica* has significant antioxidant and antidiabetic properties (Eviana R. et al., 2023).

**FIGURE 1:**
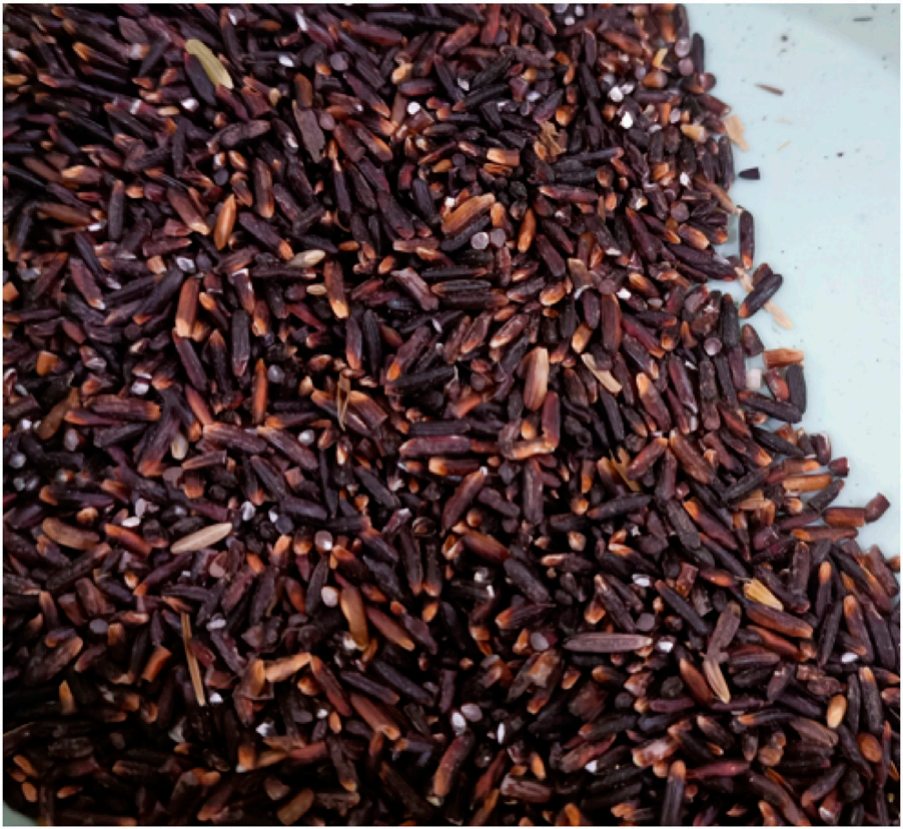
Image of *Oryza sativa L. indica.*

*Oryza sativa L. indica is* a common primary food crop for South-Asian people, commonly known as black rice (Pang et al., 2018) (Figure 1). This pigmented rice variety is known for its low fat and higher anthocyanin contents. It was only consumed by royalty in Asian nations due to its nutritional value (Panda et al., 2022). Many health benefits are related to *O. sativa L. indica* consumption as it has anti-inflammatory, anticancer, detoxifying, antimicrobial, antiviral, analgesic, and antioxidant properties (Hartati et al., 2017). Verma and Srivastav (2020) reported that *O. sativa L. indica* contains many classes of compounds such as anthocyanins (mostly cyanidin-3-glucoside and peonidin-3-glucoside), phenolic (mostly gallic and vanillic acid), and flavonoids (tricin, quercetin, and kaempferol) in adequate amounts (Verma and Srivastav, 2020). Due to the presence of these bioactive compounds, *O. sativa L. indica* has been declared as functional food (Yonata and Pranata, 2024). The presence of bioactive constituents in *O. sativa L. indica* has led to drug discovery as these constituents have great therapeutic potential as medicine**.** As our research includes plant-based bioactive constituents, we aim to overcome the side effects related to use of traditional medications such as gastrointestinal symptoms, risk of bone cancer, hypoglycemia, and weight gain.

Therefore, this work is aimed at finding the appropriate bioactive compounds from *O. sativa L*. *indica* against T2DM via “computer-aided drug designing” (CADD), which will aid in the development of novel and targeted therapeutics.

## 2 Materials and methods

### 2.1 Sample collection

*Oryza sativa L. indica* was collected from the PARC-National Agriculture Research Center (NARC), Islamabad, Pakistan. The accession number (00042662) was provided by NARC, Islamabad, Pakistan.

### 2.2 Preparation of the extract of *Oryza sativa L. indica*

*O. sativa L. indica* was sun-dried for 2 days and then de-husked. It was crushed to powder with a blender and then stored in an air-tight jar until further use (Gangga and Aini, 2018). For the extraction procedure, the maceration method was followed from Ch’ng et al. (2017) with little modifications (Mousavi et al., 2022). Ten grams of the sample powder was added in 100 mL of 70% ethanol (1:10) for the ethanol extract and 100% water for the aqueous extract (Gangga and Aini, 2018). The bottle was kept at room temperature in a mechanical shaker for 24 h. Whatman paper No.1 was used to filter the extract, and it was then stored at 4°C.

### 2.3 *In vitro* evaluation of therapeutic bioactive compounds of *Oryza sativa L. indica*

#### 2.3.1 Antioxidant property assessment

DPPH (2, 2-diphenyl-1-picrylhydrazyl) assay: this protocol was followed from Shen et al., 2010. DPPH of 0.1 mM concentration was prepared in methanol. The extract was prepared in different concentrations: 10, 30, 50, 70, and 90 μg/mL. A volume of 3 mL of the extract was added to 1 mL of the DPPH solution. The solutions were mixed thoroughly and kept at ambient temperature for 30 min. The absorbance of the solutions was calculated at 517 nm by using a UV–vis spectrophotometer. Ascorbic acid was taken as the positive control in the DPPH assay. If the absorbance of the solution is lower, it means that it has higher free radical scavenging activity (Rehman et al., 2023). The DPPH radical scavenging activity was calculated by the following formula:

$$\text{DPPH scavenging effect }\left(\%\text{ inhibition}\right)=\left\{\left(\text{Absorbance of control }–\text{Absorbance of sample}\right)/\text{Absorbance of control})*100\right\}.$$

#### 2.3.2 Antidiabetic assay

Alpha amylase assay: the alpha amylase assay was carried out following Pandey and Sharma (2022) with little modifications. The reaction mixture contained 1 mL of alpha-amylase and 1 mL of the extract. The mixture was then kept in an incubator at 37°C for 10 min. Then, 1 mL of the starch solution (1% v/v) was added and kept in an incubator at 37°C for 15 min. DNSA reagent (2 mL) was added, which stopped the reaction. The mixture was then transferred to a boiling water bath for 5 min and then cooled to room temperature. Acarbose was taken as the positive control. The extract and acarbose were prepared in different concentrations: 10, 30, 50, and 70 μg/mL. The absorbance of the mixture was calculated at 546 nm by using a UV–vis spectrophotometer. The extract was absent in the control process; therefore, it represented 100% activity. The inhibition percentage of alpha-amylase by the extract and acarbose can be calculated as follows:

$$\left(\%\text{ inhibition}\right)=\left\{\left(\text{Absorbance of control }–\text{Absorbance of sample}\right)/\text{Absorbance of control})*100\right\}.$$

#### 2.3.3 Statistical analysis

The statistical analysis of data was carried out using the linear regression technique using GraphPad Prism software.

### 2.4 *In silico* analysis of bioactive constituents in *Oryza sativa L. indica* and its potential targets against T2DM

#### 2.4.1 Gas chromatography–mass spectrometry (GC–MS) profiling

The Shimadzu QP-2020 system with the SH-Rxi-5Sil mass spectrophotometer was used for analysis, and it was present in the department of USPCASE, NUST. The column specifications were 30 m × 0.25 mm ID × 250 μm df, and helium was used as the carrier gas with 1 mL/min of constant flow (Hu M. et al., 2023). One microliter of the extract solution was injected, and the injection port temperature was kept at 200°C in a split ratio of 10:1. The column temperature was held for 1 min at 40 °C and then increased to 280°C and held for 5 min. The MS-ion source was used with the following conditions: temperature of 200°C, electron energy of 70 eV, interface temperature was kept at 200°C, and scan range was 35–800 m/*z*. The constituents were identified by paralleling their relative abundances and mass spectra and compared with the library of NIST.

#### 2.4.2 *In silico* pharmacokinetic and toxicity analysis of phytocompounds

Predictions based on pharmacokinetic characteristics can be helpful for understanding the molecular mechanisms of compounds. Therefore, it is important to evaluate their ADMET properties (Chikowe et al., 2024).

A total of 184 phytochemicals were retrieved from the GC–MS data of the ethanol extract of *O. sativa L. indica*. These compounds were subjected to online ADMET screening tools. Drug parameters including Lipinski’s rule of 5 and toxicity parameters (hERG, hepatotoxicity, AMES toxicity, carcinogenicity, and acute toxicity rule), human intestinal absorption (HIA positive), CYP450, molecular refractivity (40–130), and TPSA (<140 Å) (Zadorozhnii et al., 2022) were used to screen bioactive compounds using SwissADME (http://www.swissadme.ch/) (Daina et al., 2017) and ADMETlab 2.0 (https://admetmesh.scbdd.com/) (Xiong et al., 2021) databases, which were accessed on 15 May 2023 and 30 May 2023, respectively (Table1). Compounds were only retained if they satisfied the ADMET criteria.

**TABLE 1:** Parameters used for screening of drug-like compounds.

S.No#	Criteria name	Range of criteria
1	Lipinski rule of 5	No violations
2	herG blockers	active
3	Hepatotoxicity	Negative
4	AMES toxicity	Negative
5	Carcinogencity	Non-carcinogen
6	Acute toxicity rule	0 alert (s)
7	HIA	HIA^+^
8	PgP substrate	Positive
9	Rotatable bonds	≤10
10	TPSA	<140 Å
11	CYP 450 substrate	Substrate
12	Molar refractivity	40–130

#### 2.4.3 Acquisition of potential targets of bioactive compounds

Three databases, Swiss Target Prediction (http://www.swisstargetprediction.ch/) (Daina et al., 2019), SuperPred (https://prediction.charite.de/) (Gallo et al., 2022), and TTD (https://db.idrblab.net/ttd/) (Zhou et al., 2022) (accessed on June 2023 and July 2023 respectively), were used to search targets for the shortlisted active compounds, and key words used for the search were *Homo sapiens* and T2DM. Target gene data were acquired by combining the targets from all sources, and duplicates were removed from the final list (Hu S. et al., 2023). UniProtKB was employed to acquire the standard names of the genes (Consortium, 2021).

#### 2.4.4 Acquisition of potential targets of T2DM

“T2DM” was used as the keyword in the target prediction databases, i.e., Gene Card (https://www.genecards.org/), Mala Card, OMIM (https://omim.org/), PharmGKB (https://www.pharmgkb.org/), therapeutic target database (TTD, https://db.idrblab.net/ttd/search/ttd/) (Zhou et al., 2022), and literature (Wang et al., 2019). We accessed for T2DM-related targets of action (Kwang et al., 2021). The final target genes of T2DM were obtained by combining data from all sources and removing the duplicate genes. In the end, the targets of bioactive constituents of *O. sativa L. indica* and targets related to T2DM were intersected using Venn plot by Venny 2.0.2 software (https://bioinfogp.cnb.csic.es/tools/venny/index2.0.2.html) to extract the overlapping targets for subsequent analysis.

#### 2.4.5 Protein–protein interaction (PPI)

The overlapping targets obtained previously were imported into the online STRING database (https://string-db.org/) (Szklarczyk et al., 2023). “*H. sapiens*” and a confidence score >0.7 were used as the screening criteria according to the methodology followed by Hu M. et al., 2023. Then, data from STRING were exported to Cytoscape (version 3.9.1) to reconstruct a network to analyze the functional interaction between the target proteins. CytoHubba plug-ins were used to screen out the core or hub targets. Then, the degree of target nodes in the network was calculated following the method from Zhai and Shu, 2021. Degree is described as the “total number of interactions of a particular node (genes target) in the network.” We screened out the nodes with highest values of degree and used them for further analysis.

#### 2.4.6 Gene Ontology (GO) and Kyoto Encyclopedia of Genes and Genomes (KEGG) enrichment analysis

Online bioinformatics databases were used to gain information about GO and biological processes (BP), cellular components (CC), and molecular functions (MF) of the hub genes. The Database for Annotation, Visualization, and Integrated Discovery (DAVID) (https://david.ncifcrf.gov/tools.jsp (Sherman et al., 2022) and KEGG pathways (http://www.genome.jp/kegg/pathway.html) were used (Kanehisa et al., 2017). The top 10 enriched terms of BP, CC, and MF were obtained from the GO analysis, and the top 15 enriched pathways were obtained from KEGG (Noor et al., 2022).

#### 2.4.7 Network construction

Different networks were constructed using the Cytoscape 3.9.1 software, such as the compound–target (C–T) network, target–pathway (T–P) network, and compound–target–pathway (C–T–P) network following the method from Liu et al., 2021.

#### 2.4.8 Compound–target protein docking

Structures of the shortlisted compounds were retrieved from the online database PubChem in the SDF format (Kim et al., 2016), and crystalline structures of proteins were retrieved from RCSB Protein Data Bank (https://www.rcsb.org/) in the PDB format (Hu S. et al., 2023). Binding pockets of proteins were retrieved through literature search. Discovery Studio 21.1.0 was used to modify the PDB structure of proteins, such as the addition of hydrogen atoms and removal of heteroatoms and water molecules. Docking was carried out by PyRx software between proteins and the shortlisted compounds (Opo et al., 2021). Docking results displayed nine binding poses, and only those docked poses having the lowest root-mean-square deviation (RMSD) and lowest binding energy were selected. The binding energy of the docked complex was used as the primary criteria for screening of candidate compounds and their targets (Gackowski et al., 2023). Discovery Studio was used to visualize the 2D and 3D interactions of the best compounds with the target proteins.

#### 2.4.9 Molecular dynamics (MD) simulations

MD simulations were performed using GROMACS 2020.1. The topology of proteins and docked ligands was made with CHARM36 force field (Ahmed et al., 2022). Complexes were immersed in a dodecahedron box of water molecules. The gmx genion module was used to add Na+ and Cl^–^ ions to neutralize the charges of the docked complex (Kausar et al., 2022). The energy of the system was minimized by a 5,000-step process. Then, the next step was the establishment of equilibrium between the ligand–protein complex at constant NVT and NPT. Finally, gmx grompp and mdrun modules were used to run 100 ns of MD simulation of the ligand–protein complex. Different modules of GROMACS, gmx rmd, gmx rmsf, gmx gyrate, gmx hb, and gmx sasa, were used to get the RMSD, RMSF, GYRATE, H-BONDS, and SASA of the docked complexes, respectively (Choudhury and V, 2023; Kausar et al., 2022). Root-mean-square deviation (RMSD) was used to measure the structural stability of the complex (Arif et al., 2021). The root-mean-square fluctuation (RMSF) was used to measure the structural elasticity of the protein (Abchir et al., 2023). Radius of gyration was used to measure the compactness of the protein. Hydrogen bond analysis was performed to investigate the stability developed by ligand–protein interactions. The more the number of hydrogen bonds, the more binding between the ligand and protein complex (Kumar et al., 2023). The solvent-accessible surface area (SASA) of a protein is a measure of the surface area of the protein that is exposed to the solvent. Proteins with more SASA have more surface area exposed to the solvent. This makes it more flexible and suitable for ligand binding.

#### 2.4.10 Biological activity identification

The online tool PASS (prediction of activity spectra for substances) was used to evaluate the molecular interaction of a small biological molecule with various biological attributes. This database has inbuilt Pa and Pi notations for active and inactive compounds with values in the range of 0.000–1.000 (Kausar et al., 2022).

## 3 Results

### 3.1 *In vitro* evaluation of the therapeutic potential of extracts of *Oryza sativa L. indica*

#### 3.1.1 Antioxidant property determination

**FIGURE 2:**
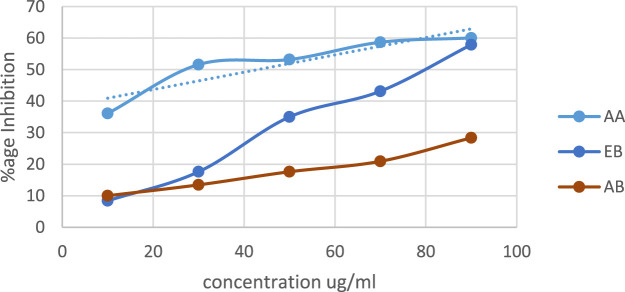
DPPH activity of aqueous and ethanol extracts of *Oryza sativa L. indica.* Ethanol extract (EB) R2 = 0.9896; aqueous extract (AB) R2 = 0.9729.

DPPH (2, 2-diphenyl-1-picrylhydrazyl) assay: the antioxidant activity of extracts of *O. sativa L. indica* was measured by calculating % inhibition. Ascorbic acid (AA) was used as the positive control. In Figure 2, linear regression shows that % inhibition increases as the concentration for samples and ascorbic acid increases. Both extracts have antioxidant activity slightly lower than that of ascorbic acid (*p*-value ≤0.05). The ethanol extract of black rice (EB) has higher antioxidant activity with a *p*-value ≤0.0005 in comparison to the aqueous extract (AB) with a *p*-value ≤0.02.

#### 3.1.2 Alpha amylase activity assay

**FIGURE 3:**
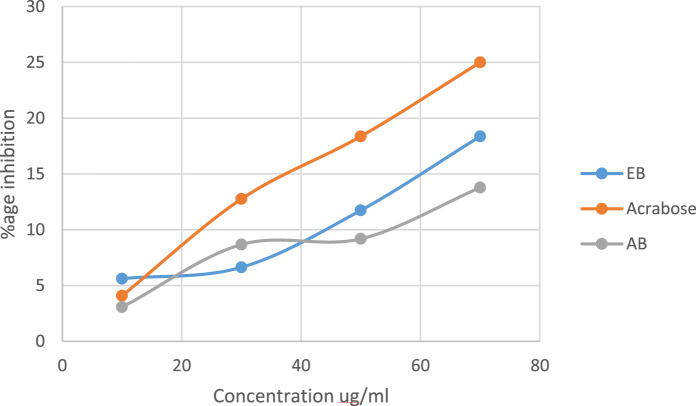
Alpha amylase activity of aqueous and ethanol extracts of *Oryza sativa L. indica.* Ethanol extract (EB) *R* ^2^ = 0.9997; aqueous extract (AB) *R* ^2^ = 0.9146.

The linear regression as shown in Figure 3 depicts the alpha amylase activity of the extracts. An increasing trend of % inhibition with increasing concentration was obtained. Acarbose was used as the control. Both extracts have alpha amylase activity slightly lower than that of acarbose (*p*-value ≤0.05). The ethanol extract of black rice (EB) has higher alpha amylase activity with a *p*-value ≤0.0001 in comparison to the aqueous extract (AB) (*p*-value ≤0.05).

### 3.2 In silico evaluation of bioactive compounds in *Oryza sativa L. indica* and its potential targets against the treatment of T2DM

#### 3.2.1 Identification and selection of active compounds in *Oryza sativa L. indica*

A total of 184 compounds were identified by GC–MS analysis, and the compound names, molecular weight, molecular refractivity, rotatable bonds, hydrogen bond donor, hydrogen bond acceptor, and MLOGP values are enlisted in Supplementary Table S1 **.** All identified compounds were subjected to ADME screening. A total of 111 compounds met the toxicity parameters, out of which 86 had followed Lipinski’s rule of 5. After that, HIA and Pgp substrate filter were applied, which screened out 63 compounds. Then, 54 compounds were screened out after using TPSA (20–140Å^2^) and rotatable bonds (10) as filters. Finally, CYP2D6, CYP3A4, and molar refractivity (40–130) parameters had shortlisted 18 compounds. These 18 compounds were regarded as bioactive compounds as they met the criteria of drug-like properties. The compounds’ name, PubChem ID, molecular mass, molar refractivity, HBA, HBD, RB, and M-LogP values of the 18 compounds are given in Table 2.

**TABLE 2:** Physiochemical properties of shortlisted bioactive compounds.

S. No#	Formula	PubChem CID	Compounds	MR	MW	RB	HBD	HBA	M-LogP
1	C9H18N2O3	259583	dl-Alanyl-dl-leucine	52	202	6	4	3	0.01
2	C8H19N	12735	2-Octanamine	43	129	5	1	1	2.22
3	C9H12FNO2	541491	Benzeneethanamine, 3-fluoro-.beta.,5-dihydroxy-N-methyl	46	185	3	4	3	1.07
4	C9H12N2O	541846	2-Methylamino-N-phenyl-acetamide	48	164	4	2	2	0.96
5	C10H15N	199116	Phenethylamine, p,.alpha.-dimethyl	48	149	2	1	1	2.49
6	C9H1302	4,782	dl-Phenylephrine	47	167	3	3	3	0.65
7	C9H13NO	214613	3-Hydroxy-N-methylphenethylamine	45	151	3	2	2	1.53
8	C11H25N	114476	1-Methyldecylamine	57	171	8	1	1	3.13
9	C9H12FNO2	127901	Benzeneethanamine, 2-fluoro-.beta.,3-dihydroxy-N-methyl	46	185	3	4	3	1.07
10	C11H20O2	5634	Undecylenic acid	56	184	9	2	1	2.76
11	C8H20N2	83131	1,6-Hexanediamine, N,N′-dimethyl	46	144	7	2	2	1.29
12	C13H21NO	71159880	N-Desmethyltapentadol	65	207	5	2	2	2.68
13	C16H24O2	544141	Nonanoic acid, 6-phenyl-, methyl ester	75	248	9	2	0	3.9
14	C11H15N5O4	56688435	1H-Purine-8-propanoic acid, .alpha.-amino-2,3,6,7-tetrahydro-1,3,7-trimethyl-2,6-dioxo-	71	281	3	6	2	−2.72
15	C11H9NO6	542066	Hydrastininic acid	57	251	4	6	2	−0.54
16	C9H17N	161455	1-(5-Bicyclo [2.2.1]heptyl)ethylamine	43	139	1	1	1	2.15
17	C15H20O4	22571182	Isophthalic acid, isobutyl propyl ester	73	264	8	4	0	3.18
18	C12H17NO2	240161	Cyclohexanecarboxamide, N-furfuryl-	58	207	4	2	1	1.22

#### 3.2.2 Target gene prediction of bioactive compounds of *Oryza sativa L. indica*

A total of 4,091 targets of the 18 bioactive compounds were identified using Swiss Target Prediction and SuperPred databases. Duplicates were removed, and a total of 924 genes were left.

#### 3.2.3 Target gene prediction of T2DM

**FIGURE 4:**
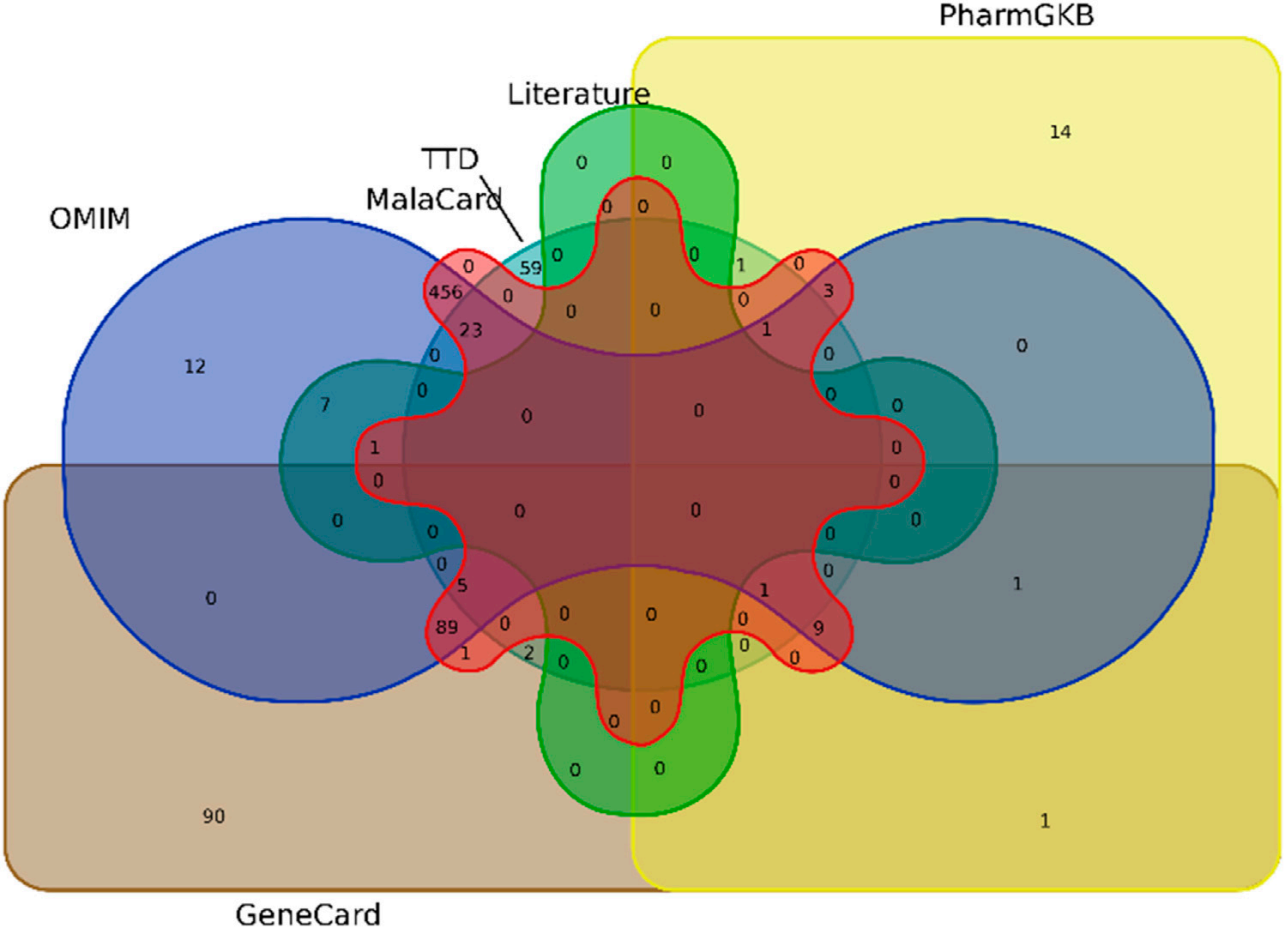
Venn diagram illustrating the overlapping targets of T2DM from six sources.

**FIGURE 5:**
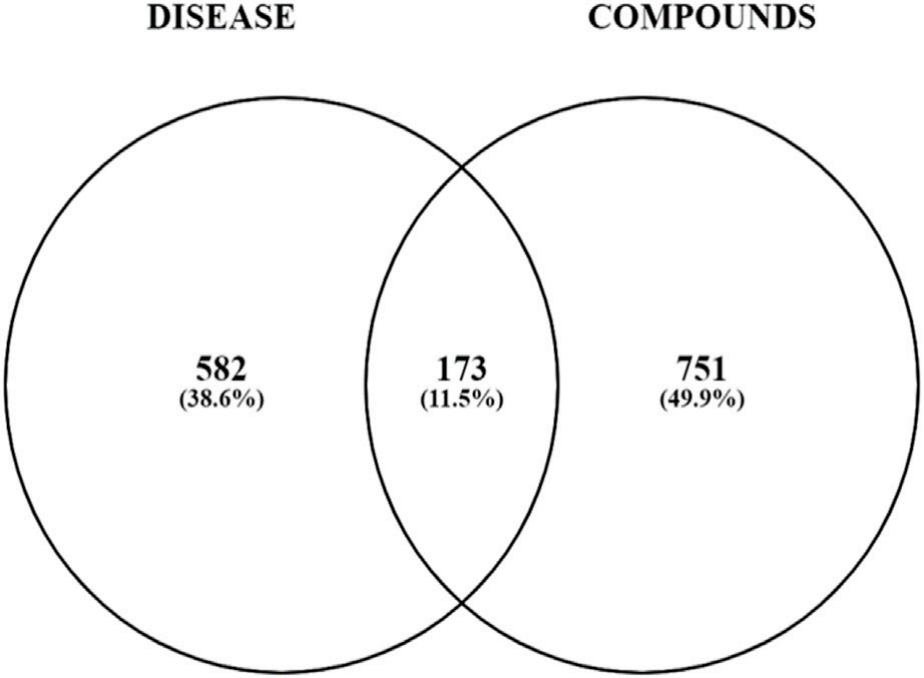
Venn diagram illustrating the overlapping targets of T2DM and compounds.

Comprehensive target mining from different databases and literature was performed. The GeneCard, MalaCard, OMIM, PharmGkb, TTD, and literature were used to acquire 199, 594, 607, 30, 91, and 8 T2DM targets, respectively, as shown by the Venn diagram in Figure 4 **.** Duplicate targets were eliminated, and thus, a total of 755 target genes related to T2DM were obtained. A total of 173 overlapping targets were acquired after the intersection of 924 targets of compounds and 755 T2DM-related targets, as shown by the Venn diagram in Figure 5.

#### 3.2.4 PPI network construction

**FIGURE 6:**
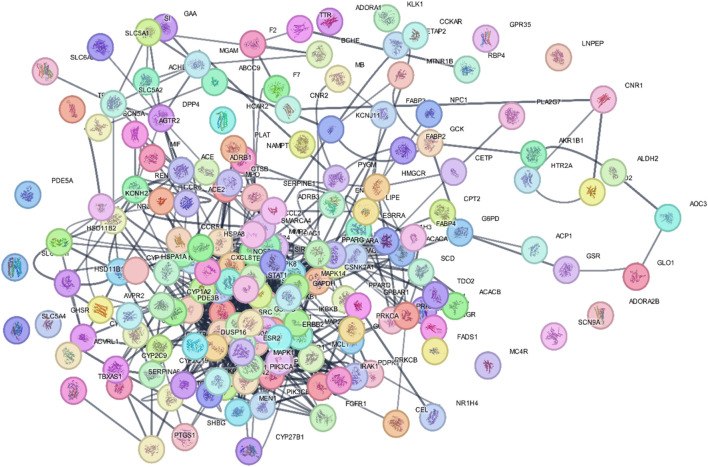
PPI network of 173 overlapping targets were constructed using STRING.

**FIGURE 7:**
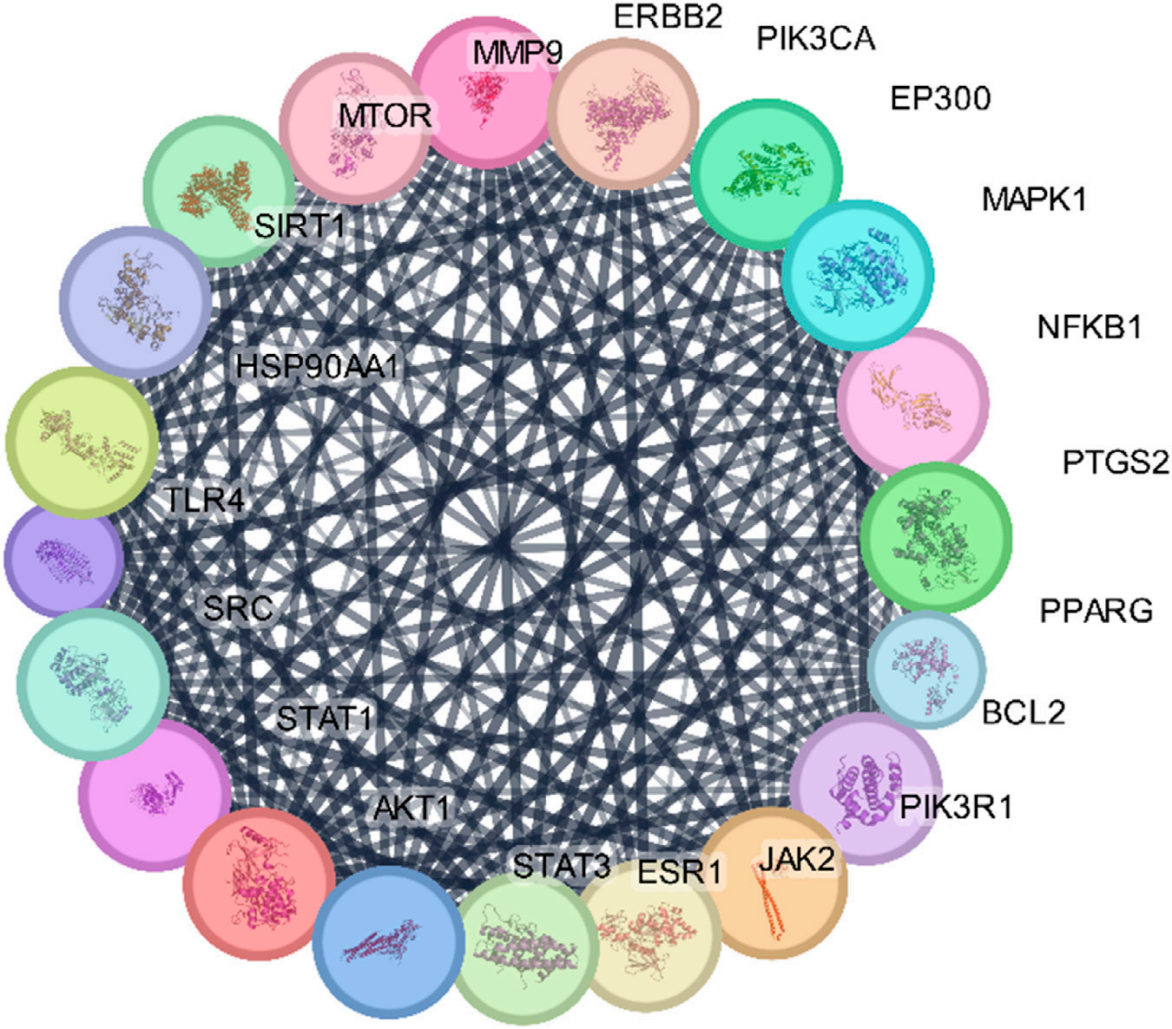
Network of top 20 hub genes with higher degree values was constructed via Cytoscape software.

**FIGURE 8:**
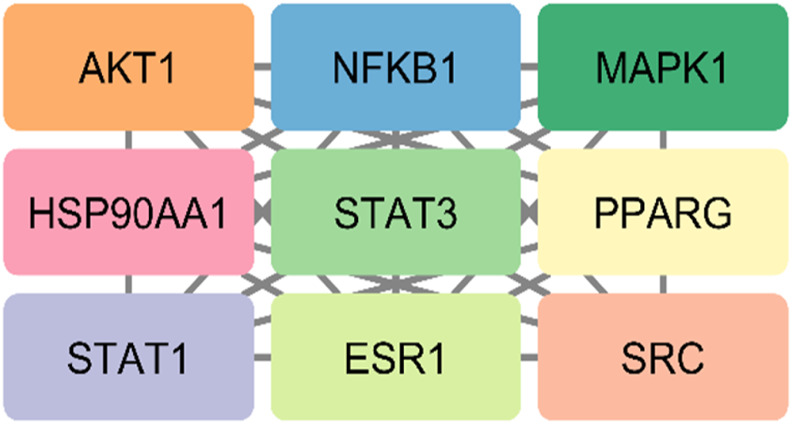
Top nine hub genes that were selected on basis of higher degree values of genes via CytoHubba.

**FIGURE 9:**
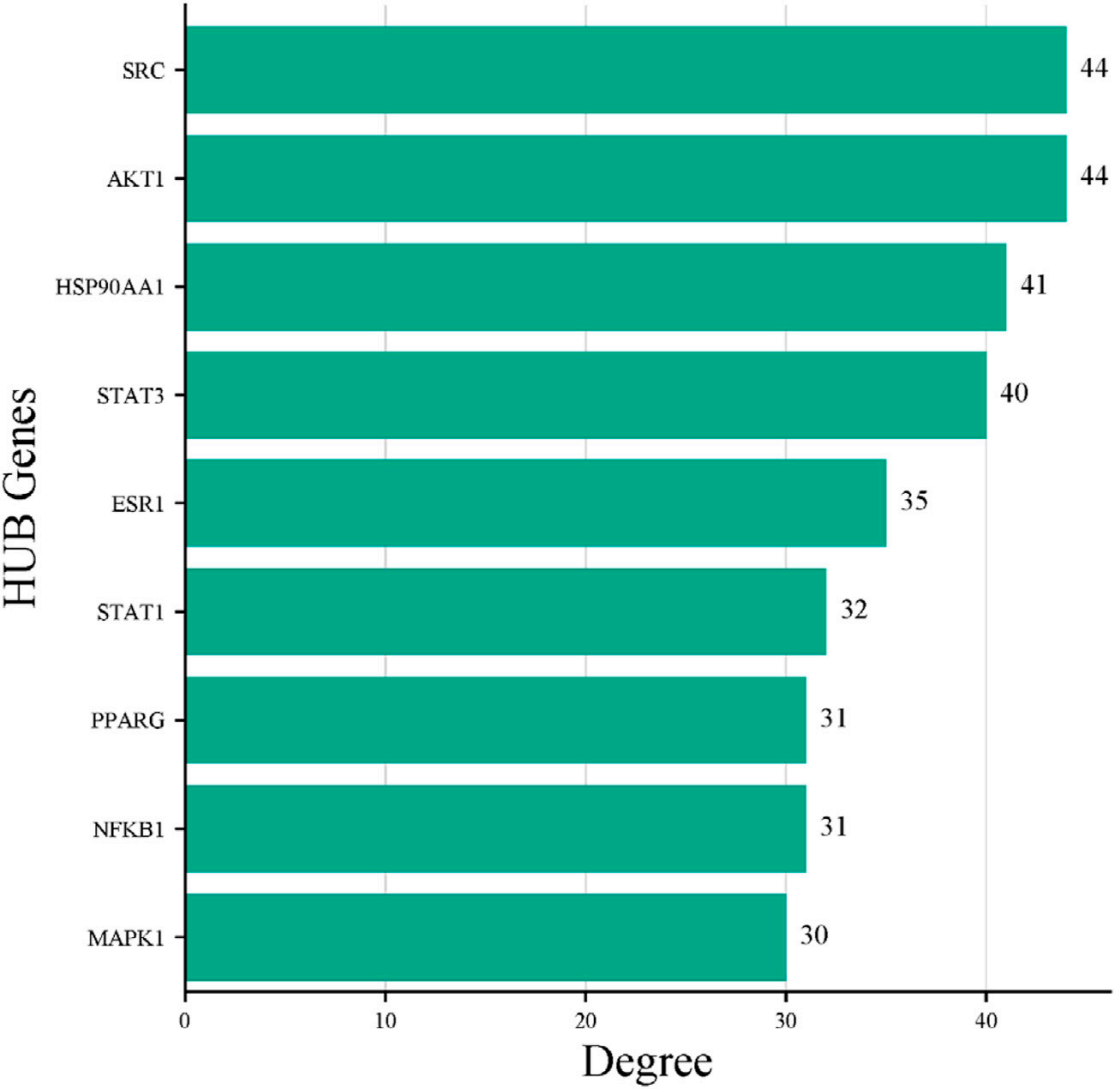
Bar plot of the nine HUB genes. *X*-axis represents degree score, and *Y*-axis represents the gene symbols.

The STRING database was used to construct the PPI network of 173 overlapping targets to illustrate the interaction between the targets of compounds related to T2DM (Figure 6). The network was exported to Cytoscape for visualization, where nodes represent genes and edges represent interactions. The CytoHubba plugin was used to determine the degree value of nodes. The first 20 genes with the highest degree value are shown in Figure 7. Only the top nine hub genes with the highest degree value in the network were selected—signal transducer and activator of transcription 3 (STAT3), heat shock protein 90 kDa alpha (HSP90AA1), AKT1, SRC proto-oncogene, non-receptor tyrosine kinase (SRC), ESR1, mitogen-activated protein kinase 1 (MAPK1), nuclear factor kappa-light-chain-enhancer of activated B cells (NFKB1), histone acetyltransferase p300 (EP300), and CREB binding protein (CREBBP)—which are considered to be the potential targets for *O. sativa L. indica* in treating T2DM (Figures 8, 9).

#### 3.2.5 Gene Ontology (GO) and Kyoto Encyclopedia of Genes and Genomes (KEGG) enrichment analysis

**FIGURE 10:**
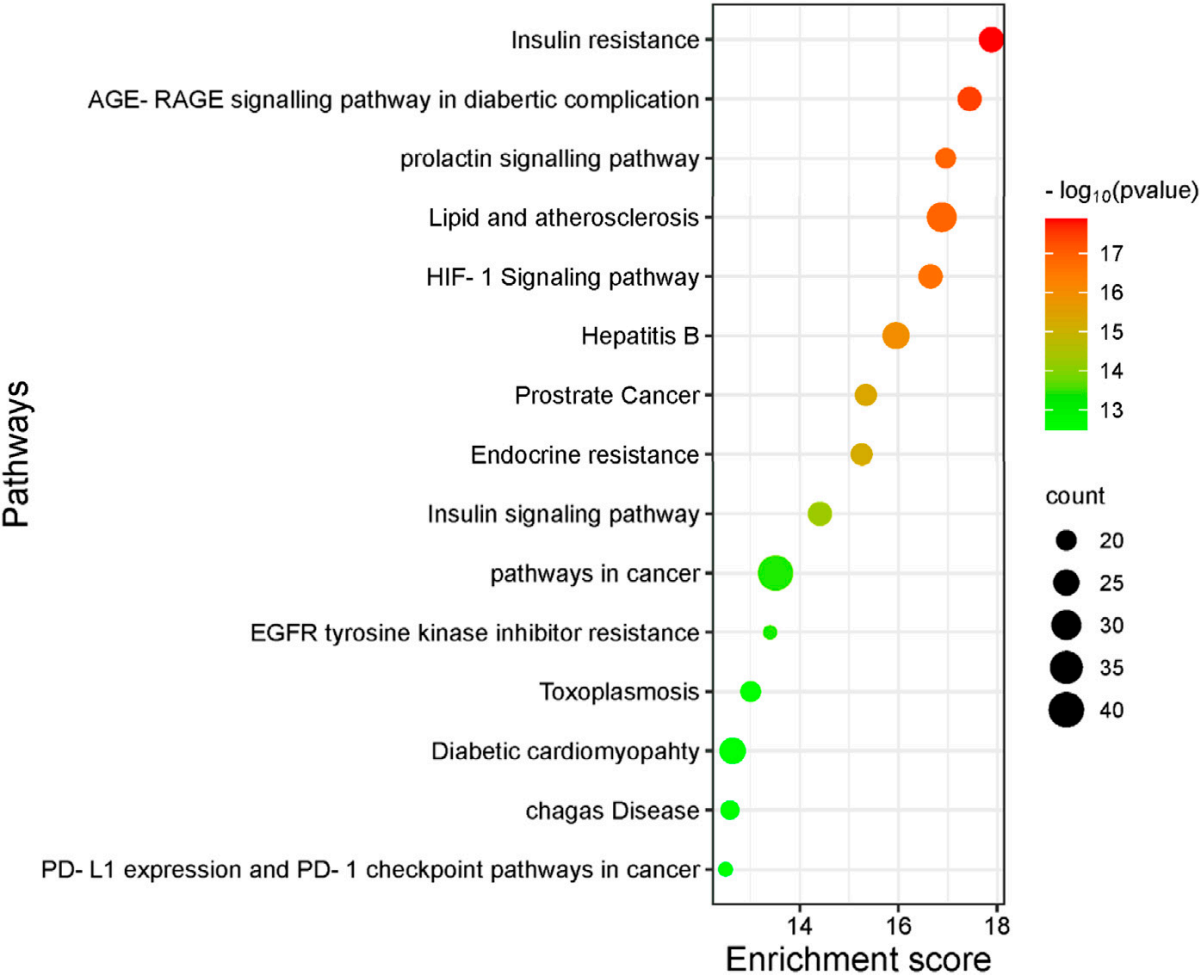
Top 15 most significantly enriched pathways are exhibited with their *p*-value and gene count. The larger the bubble is, the greater the number of enriched genes in the signaling pathways.

**FIGURE 11:**
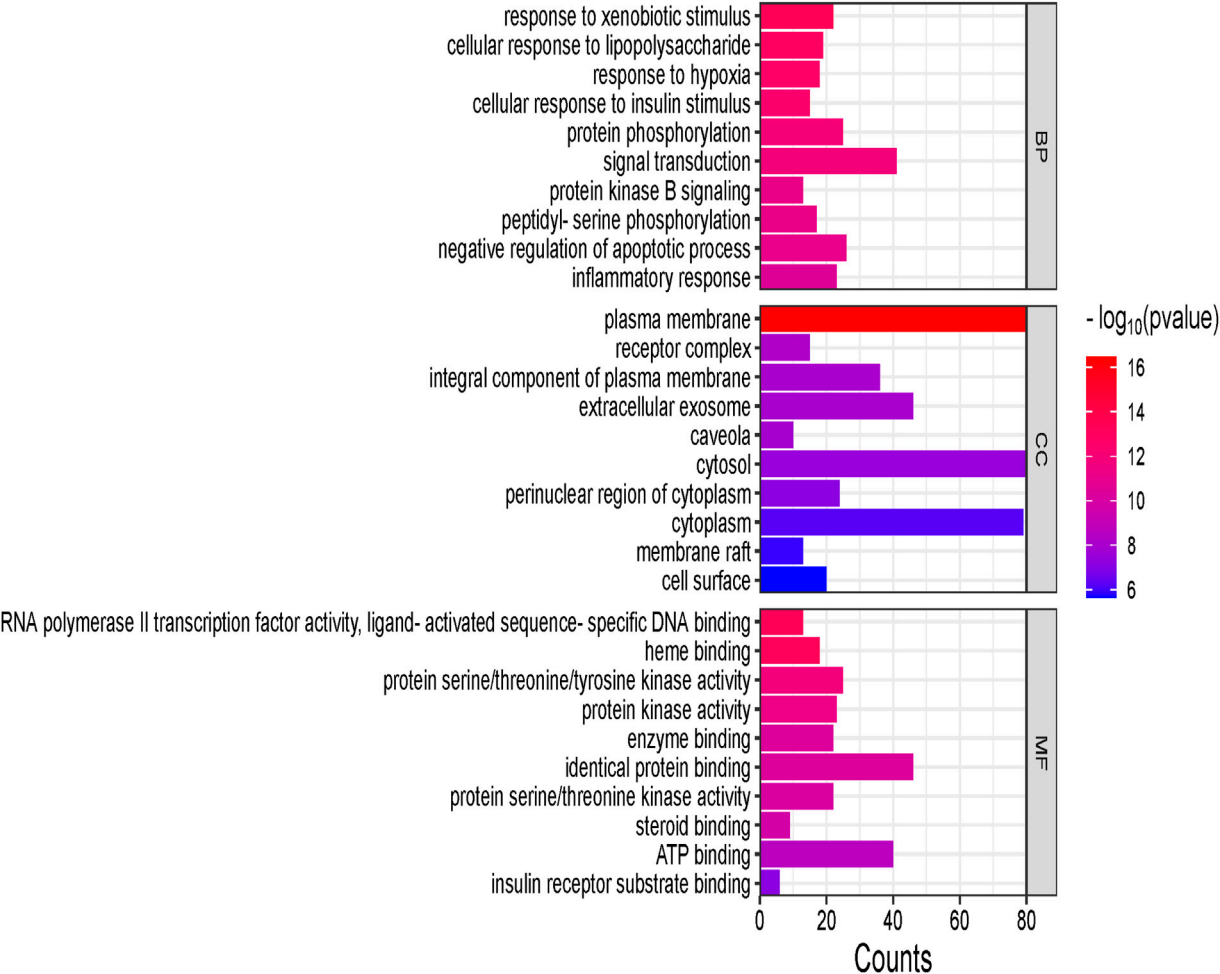
Biological processes (BP), cellular components (CC), and molecular functions (MF) are shown in the vertical axis, whereas gene counts are shown in the horizontal axis. The larger the line suggests, the more genes are enriched to biological processes, cellular components, and molecular functions.

The GO function enrichment analysis was conducted using the DAVID database. The top 15 significant pathways are shown along with their *p*-value and gene count in Figure 10, which are insulin resistance, AGE-RAGE signaling pathways in diabetic complication, lipid and atherosclerosis, and insulin signaling pathway. These pathways have a strong correlation with the pathogenesis of T2DM. Top 10 BP, CC, and MF related to genes are exhibited in Figure 11.

#### 3.2.6 Network construction

**FIGURE 12:**
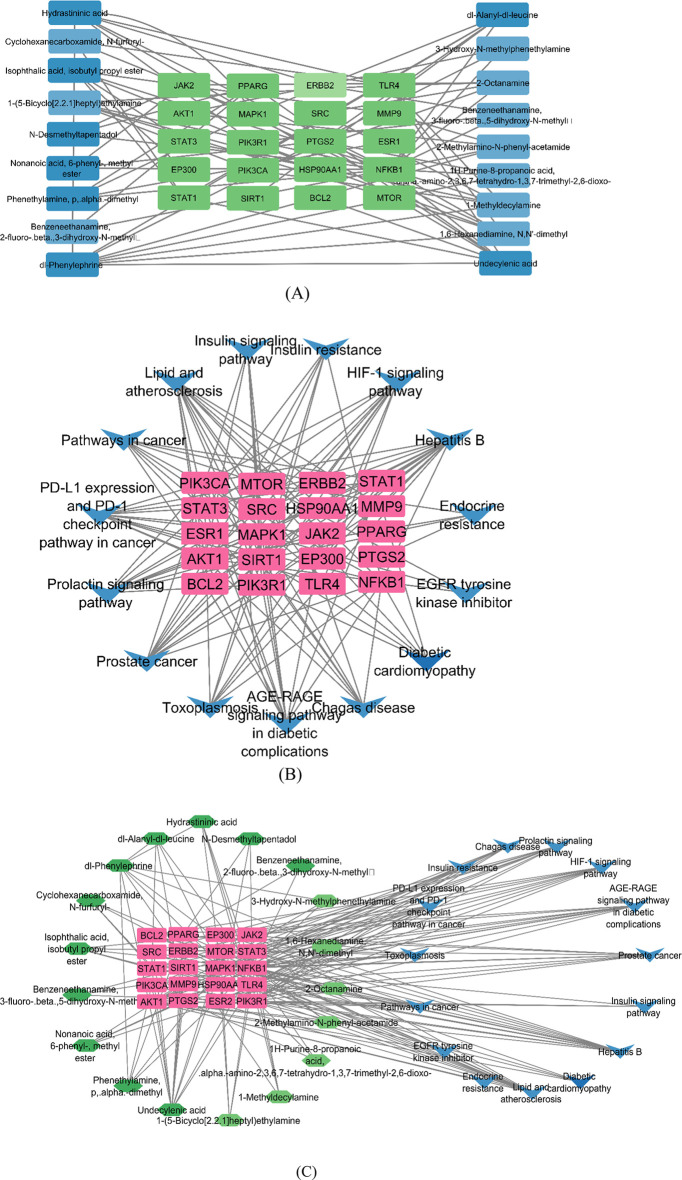
Network construction by Cytoscape. **(A)** Compound–target network, where the green nodes are targets and the blue nodes are compounds. **(B)**Target–pathway network, where the pink nodes represent targets and the blue nodes represent pathways. **(C)** Compound–target–pathway network, where the green, pink, and blue nodes represent the compounds, targets, and pathways, respectively.

Compound–target (C–T), target–pathway (T–P), and compound–target–pathway (C–T–P) networks were constructed using the Cytoscape 3.9.1 software, as shown in Figure 12. In the pictorial representation of the network, nodes represent targets, compounds, and pathways, and the edges represent C–T, T–P, and C–T–P interactions.

#### 3.2.7 Molecular docking

**FIGURE 13:**
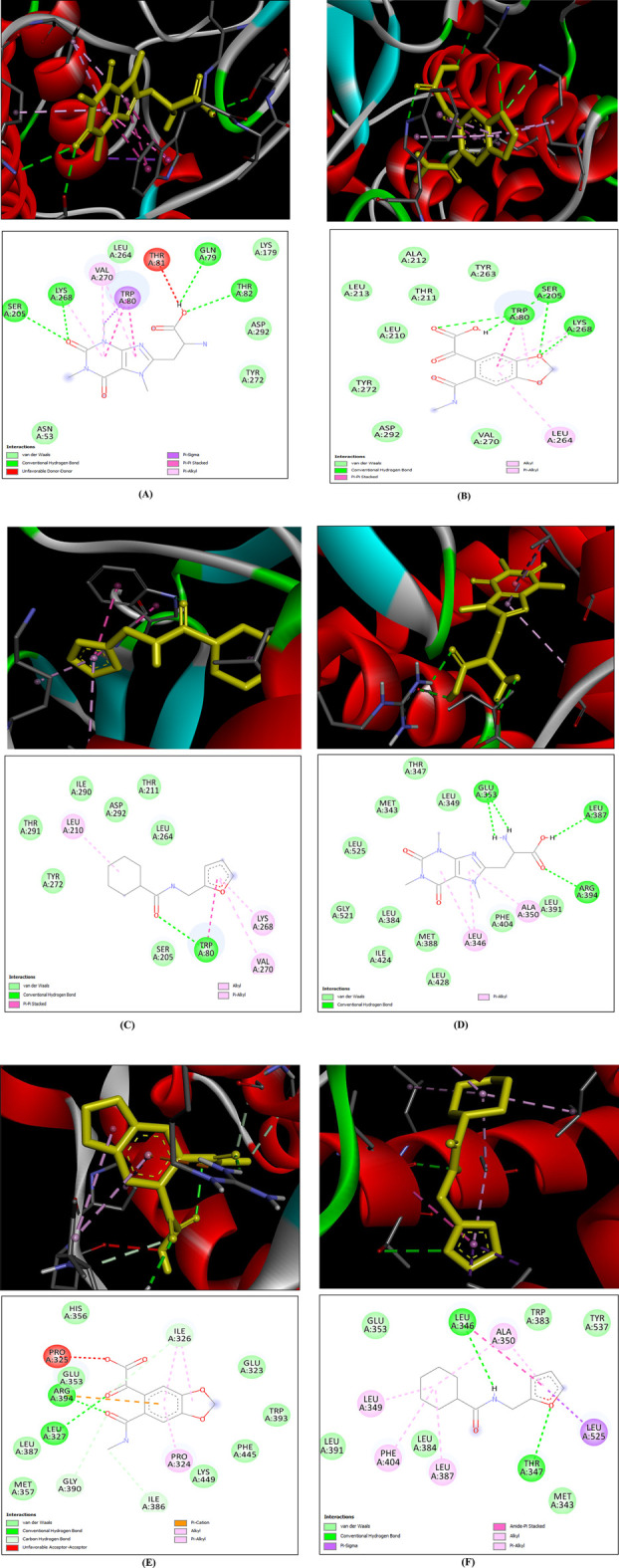
3D (top) and 2D (bottom) structures of ligand bound to protein within its active site. In the 3D structure, the yellow-colored structure represents the ligand, the red ribbon structure represents the protein, and the dotted lines are different bond types between the ligand and protein. In the 2D structure, the circle represents the residues of binding site of protein, the gray line structure represents the ligand, and the different colored doted lines represent different bond types between ligand and residues of protein **(A)** AKT1–C14, **(B)** AKT1–C15, **(C)** AKT1–C18, **(D)** ESR1–C14, **(E)** ESR1–C15, and **(F)** ESR1–C18.

**FIGURE 14:**
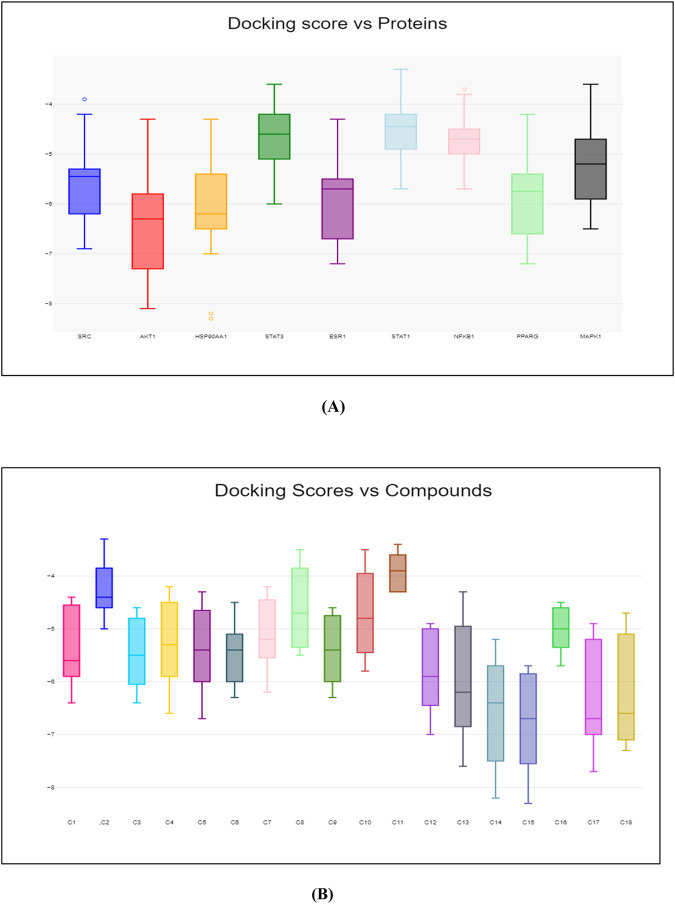
Box plot of binding score. **(A)** *Y*-axis represents the binding energy, while *X*-axis represents the name of proteins. **(B)** *Y*-axis represents the binding energy, while *X*-axis represents the number of compounds.

Molecular docking was carried out between the compounds and target proteins to investigate the interacting binding energy (Choudhury et al., 2024). Binding energies of the docked complexes and their PDB ID are shown in Table 3. AKT1 and ESR1 gave the lowest binding energy with compounds 14, 15, and 18, which indicated that these were stable complexes. The docked poses of the complexes were visualized using Discovery Studio and are depicted in Figure 13. C14 formed hydrogen bonds with residues Gln79, Thr82, Ser205, and Lys268 of the AKT1 protein. Similarly, it formed hydrogen bonds with residues Glu353, Leu387, and Arg394 of the ESR1 protein. C15 formed hydrogen bonds with residues Trp80, Ser205, and Lys268 of the AKT1 protein. Similarly, it formed hydrogen bonds with residues Ile326, Ile386, Gly390, and Arg394 of ESR1 protein. C18 formed hydrogen bonds with the binding pocket residue Trp80 of the AKT1 protein. Similarly, C18 formed hydrogen bonds with residues Thr347 and Leu346 of ESR1. According to box plot interpretation, compounds 14, 15, and 18 and proteins AKT1 and ESR1 were selected for MD simulations, as shown in Figure 14.

**TABLE 3:** Binding scores are shown against target proteins and bioactive compounds in kcal/mol.

Compound No.	SRC (1Y57)	AKT1 (3O96)	HSP90AA1 (7KRJ)	STAT3 (6NJS)	ESR1 (1R5K)	STAT1 (1BF5)	NFKB1 (1NFI)	PPARG (3E00)	MAPK1 (1WZY)
C1	−5.6	−6.1	−6.4	−4.5	−5.7	−4.4	−4.6	−5.6	−4.9
C2	−4.2	−5	−4.4	−3.8	−4.7	−3.3	−4.5	−4.4	−3.9
C3	−5.5	−6.4	−6.3	−4.6	−5.7	−4.7	−4.9	−5.8	−5.2
C4	−5.3	−5.8	−6	−4.2	−5.5	−4.4	−4.6	−6.6	−5.2
C5	−5.4	−6.7	−5.7	−4.6	−5.9	−4.3	−4.7	−6.1	−5.1
C6	−5.4	−6.3	−6.1	−5	−5.9	−4.5	−5.3	−5.5	−5.2
C7	−5.2	−6.2	−5.4	−4.5	−5.6	−4.2	−4.4	−5.5	−5
C8	−5.5	−5.5	−4.7	−3.9	−5.2	−3.5	−3.8	−5	−4.1
C9	−5.4	−6.3	−6.3	−4.7	−5.7	−4.6	−4.8	−5.7	−5.4
C10	−5.4	−5.8	−4.8	−4.2	−5.5	−3.5	−3.7	−5.4	−4.6
C11	−3.9	−4.3	−4.3	−3.6	−4.3	−3.4	−3.7	−4.2	−3.6
C12	−6.2	−7	−6.5	−5.1	−6.4	−4.9	−4.9	−5.9	−5.6
C13	−6.2	−7.6	−6.5	−4.9	−6.8	−4.3	−5	−6.9	−5.9
C14	−6.9	**−8.1**	−8.2	−5.7	**−6.9**	−5.2	−5.7	−6.4	−6.3
C15	−6.7	−8.1	−8.3	−6	−6.7	−5.7	−5.7	−7	−6.5
C16	−5.1	**−5.3**	−5.4	−4.5	**−5.7**	−4.9	−4.5	−5	−4.7
C17	−6.7	−7.7	−6.7	−5.2	−6.8	−4.9	−5.2	−7.2	−6
C18	−6.6	**−7.3**	−7	−5.1	**−7.2**	−5.1	−4.7	−7	−6.1

SRC, Proto-oncogene tyrosine-protein kinase; AKT1, AKT serine/threonine kinase 1; HSP90AA1, Heat shock protein 90 alpha family class A member 1; STAT3, Signal transducer and activator of transcription 3; ESR1, Estrogen receptor 1; STAT1, Signal transducer and activator of transcription 1; NFKB1, Nuclear factor NF-kappa-B p105 subunit; PPARG: Peroxisome proliferator-activated receptor gamma; MAPK1, Mitogen-activated protein kinase 1.

Bold values indicate the gene symbols referenced from online GenBank NCBI database.

Bold values in brackets indicate the protein ID (identifiers) for the genes taken from online Protein databank (PDB ID).

#### 3.2.8 Molecular dynamics (MD) simulations

**FIGURE 15:**
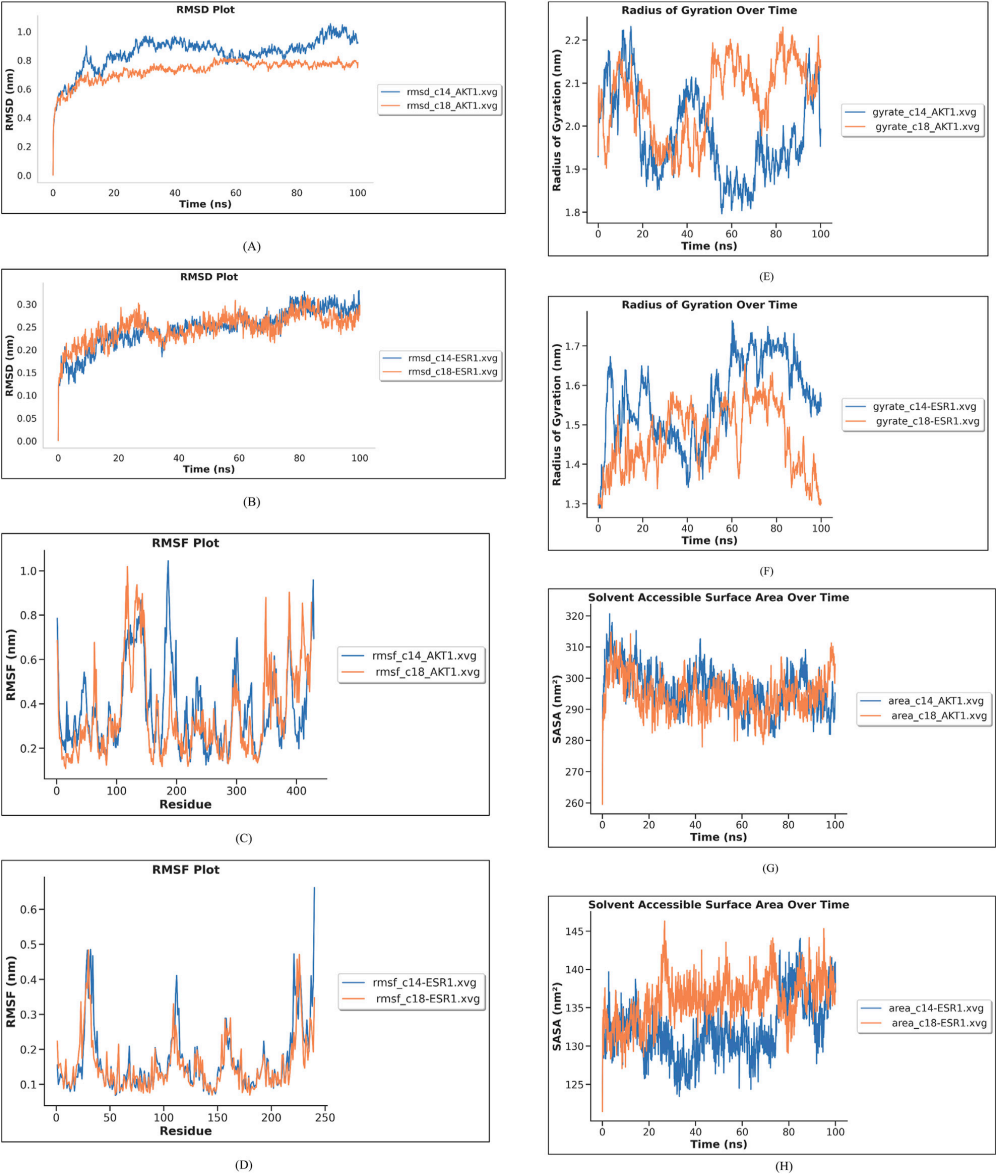
Molecular dynamic simulation results. **(A)** RMSD of compounds C14 and C18 with AKT1 is shown in blue and red-colored lines in the graph. **(B)** RMSD of compounds C14 and C18 with ESR1 is shown in blue and red lines in the graph. **(C)** RMSF of compounds C14 and C18 with AKT1 is shown in blue and red lines in the graph. **(D)** RMSF of compounds C14 and C18 with ESR1 is shown in blue and red lines in the graph. **(E)** Radius of gyration of C14 and C18 with AKT1 is shown in blue and red lines in the graph. **(F)** Radius of gyration of C14 and C18 with ESR1 is shown in blue and red lines in the graph. **(G)** SASA of C14 and C18 with AKT1 is given in blue and red lines in the graph **(H)** SASA of C14 and C18 with ESR1 is given in blue and red lines in the graph.

Based on docking, six complexes (AKT1 with C14, C15, and C18 and ESR1 with C14, C15, and C18) were selected for simulation. MD simulation was run for 100 ns to analyze the stability of the complexes and investigate the interaction between the selected ligand and protein. After running MD simulation for 100 ns, the structures of AKT1 and ESR1 proteins were found to be stable. The binding of C14 and C18 to the AKT1 and ESR1 proteins was relatively stable as compared to that of C15, and the ligands remained at the predicted binding site in the process of MD simulation. RMSD, RMSF, hydrogen bond analysis, radius of gyration, and SASA of complexes were calculated and are shown in Figure 15. RMSD and RMSF graphs were generated to investigate fluctuations in the complexes. The docked poses of compounds C14 and C18 with AKT1 and ESR1 have shown less fluctuation as compared to those of C15 with AKT1 and ESR1. RMSD of C14-AKT1, C18-AKT1, C14-ESR1, and C18-ESR1 complexes were considered in the equilibrium state due to its low fluctuation in the range of 0.1 nm–0.8 nm throughout the time of simulation, as shown in Figures 15A, B.

The root-mean-square fluctuation (RMSF) was used to evaluate the flexibility of amino acid residues of the protein. RMSF of binding pocket residues of ESR1 and AKT1 with their respective ligands (C14 and C18) showed fluctuation between 0.2 and 0.6 nm, which indicated that the protein was stabilized after docking (Figures 15C, D). All active site binding residues have shown minimum fluctuation, which means these complexes have attained stability.

Radius of gyration (Rg) is calculated as the root-mean-square distance from atoms of the protein backbone in nm against MD run time. If the radius of gyration decreases, it means that macromolecules are tightly packed and *vice versa*. Therefore, the lower the Rg values of the protein–ligand complex, more stability and less disorder in the system. Complexes C14–AKT1 and C18–AKT1 have attained a stable Rg trajectory between 1.8 and 2.2 nm, as shown in Figure 15E, and complexes C14–ESR1 and C18–ESR1 have attained a stable Rg trajectory between 1.3 and 1.7 nm, as shown in Figure 15F.

Solvent-accessible surface area uncovers the protein’s interactable surface to the solvent molecules. Complexes C14–AKT and C18–AKT1 have shown an average SASA of 300 nm^2^ (Figure 15G). The average value of SASA for complexes C14–ESR1 and C18–ESR1 was found to be 135 nm^2^ (Figure 15H). The SASA findings exhibit that internal residues of AKT1, upon binding with C14 and C18, were more accessible by the solvent as compared to ESR1 upon binding with C14 and C18.

**FIGURE 16:**
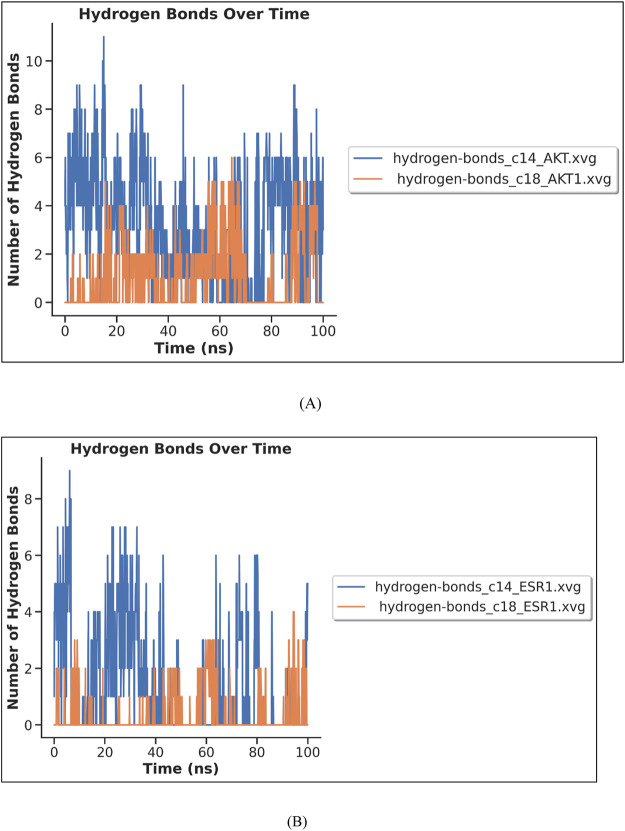
MD simulation results (H-bond analysis) of complexes: **(A)** C14–AKT and C18–AKT1 **(B)** C14–ESR1 and C18–ESR1.

Hydrogen bond analysis was carried out to measure the stability of complex interactions. The number of hydrogen bonds in each complex is shown in Figure 16. The complex C14–AKT1 formed approximately ten H-bonds during MDS, of which eight remained intact throughout the simulation. The complex C18–AKT1 formed five intact H-bonds throughout the simulation, as shown in Figure 16A. C14–ESR1 formed seven H-bonds, of which five remained intact throughout the simulation. The complex C18–ESR1 formed four H-bonds that remained intact throughout simulation, as shown in Figure 16B.

#### 3.2.9 Biological activity identification

Identification of molecular interactions between compounds and their targets is important in *in silico* and toxicity analysis. The PASS tool uses structure–activity relationship analysis (SAR) to predict approximately more than 3,000 various biological targets; hence, it reduces the possibility of failure while conducting *in vitro* and wet-lab testing. The PASS tool has shown the interaction of C14 and C18 with 843 and 631 different biological targets, respectively.

The insulin promoter in terms of Pa and Pi values for C18 (0.444, 0.045) was found more compared to that of C14 (0.326 0.112). Moreover, C18 (0.319, 0.156) and C14 (0.402, 0.103) have also shown reasonable inhibition activity against beta-glucosidase. Apart from the above targets, antiviral, anti-neurogenic, antihypertensive, and antineoplastic effects of all ligands were also given.

## 4 Discussion

T2DM is the most common metabolic disease, and it is seriously affecting peoples’ lives across the globe (Noor et al., 2022). Many therapeutic drugs are available in the market, but they only relieve the symptoms and, in addition, cause many adverse effects, mainly gastrointestinal issues, weight gain, and hypoglycemia. However, it has been reported that natural drugs derived from plant sources do not cause adverse effects (Tran et al., 2020).

Research on plant-based therapeutics is emerging as researchers are looking for novel and safer therapeutic drugs against many diseases (Halayal et al., 2023). It has been reported that medicines from different plant extracts have been used to treat T2DM (Tran et al., 2020). In another study, it was reported that *O. sativa L. indica* (black rice) possess anti-oxidant, anti-obesity, anti-inflammatory, and immune-regulatory effects (Fatchiyah et al., 2020). Black rice contains bioactive substances such as anthocyanin, flavonoids, phenolic compounds, and pro-anthocyanins, which can act as antidiabetic agents via lowering blood glucose level and increasing the insulin level (Eviana Rafika et al., 2023). The goal of the study is to explore the therapeutic potential of candidate compounds of *O. sativa L. indica* against protein targets of T2DM; therefore, this study employed the network pharmacology strategy.

In the present study, DPPH inhibition activity and alpha amylase inhibitory activity assay was performed on plant extracts to evaluate the antioxidant and antidiabetic potential. The ethanol extract showed significant antioxidant and anti-diabetic potential compared to the aqueous extract. Similar results of *in vitro* analysis have been reported in other studies (Thepthanee et al., 2021; Wongsa, 2019). In *in silico* studies, bioactive compound screening, acquisition of targets, and pathway enrichment analysis, network pharmacology analysis of bioactive compounds of *O. sativa L. indica* was carried out. A total of 184 compounds were identified by GC–MS, and 18 compounds were yielded by ADMET screening (Hartati, 2021). A total of 924 targets related to shortlisted compounds and 755 targets associated with T2DM were identified, and 173 overlapping targets were obtained from the interaction of the targets of *O. sativa* identified compounds and T2DM. These target genes were significantly enriched in several pathways related to the pathogenesis of T2DM. Nine hub genes with the highest degree score were selected: STAT3, HSP90AA1, AKT1, SRC, ESR1, MAPK1, NFKB1, EP300, and CREBBP. These genes were considered to be the potential targets of T2DM (Kwang et al., 2021). The same network pharmacology approach has been reported previously (Liu et al., 2021). Moreover, molecular docking results showed that C14, C15, and C18 exhibited good binding affinity with hub targets ESR1 and AKT1, and the docking protocol was followed from a previously reported method (Xiang et al., 2022).

It was reported in a study that C18 has been identified as an agent that binds to the S1P4 receptor, and S1P has a role in the pathogenesis of diabetes. S1P and its related molecules can increase insulin sensitivity of cells and protect β cells, therefore having the potential to be developed as drugs for T2DM (He et al., 2021). AKT1 and ESR1 have a role in the formation of metabolic diseases such as diabetes and obesity. Miao et al. (2022) have mentioned that AKT1 regulates glucose and lipid metabolism through three downstream substrates: TSC2, GSK3, and FOXO transcription factors. ESR1 inhibition can lead to development of the metabolic syndrome (MetS) caused by impaired fatty acid oxidation and impaired insulin sensitivity; thus, this shows the critical role of ESR1 in metabolic homeostasis (Miao et al., 2022).

MD simulation was carried out to gain a deeper insight into binding of compounds to their targets, and the MD simulation protocol was followed from a previous study (Ilyas et al., 2022). Four complexes, C14–AKT1, C14–ESR1, C18–AKT1, and C18–ESR1, were stabilized in 100-ns simulations and have very little fluctuation in complex formation. This study suggests that C14 and C18 might be used as potential therapeutic drugs against the targets of T2DM.

The discovery of potential bioactive substances that halt the pathogenesis of T2DM will be the defining feature of this era. Our study has shown that the active compounds C14 and C18 from *O. sativa L. indica,* both targeting ESR1 and AKT1, can significantly influence diabetes pathogenesis by the regulation of pathways involved in glucose metabolism and insulin signaling. For example, inhibitors of AKT1 may improve glucose uptake by affecting insulin signaling pathways, while ESR1-targeting drugs could affect glucose metabolic processes. These findings indicated that bioactive constituents from plant sources are potential agents against T2DM to address the disease’s underlying mechanism. However, how do these compounds modulate the insulin signaling pathways and glucose metabolism? In addition, what will be the long-term effect of these compounds targeting ESR1 and AKT1? These questions still need to be investigated. Therefore, it is suggested that further *in vitro* and wet-lab experimentations should be carried out to investigate the effective treatment against the targets of T2DM.

## 5 Conclusion

This study analyzed the potential molecular biological mechanisms of *O. sativa L. indica* in the treatment of T2DM through network pharmacology and molecular docking. *In vitro* analysis showed that extracts of *O. sativa L. indica* have good anti-inflammatory and anti-diabetic properties. The GC–MS results showed that the active components of *O. sativa L. indica* included 184 compounds, among which 18 were shortlisted on the basis of ADMET profiling. Moreover, nine hub targets were screened, which were STAT3, HSP90AA1, AKT1, SRC, ESR1, MAPK1, NFKB1, EP300, and CREBBP. Docking and MD simulations revealed that targets AKT1 and ESR1 strongly bound with C14 and C18. Therefore, we can conclude that these compounds from *O. sativa L. indica* can be used for the treatment of T2DM. However, certain questions remain to be addressed, such as how these compounds modulate the insulin signaling pathways and glucose metabolism? In addition, what will be the long-term effect of these compounds targeting ESR1 and AKT1? Therefore, experimental validation of the predicted compounds is required to assess the effective treatment against targets of T2DM.

## Data Availability

The datasets presented in this study can be found in online repositories. The names of the repository/repositories and accession number(s) can be found in the article/Supplementary Material; further inquiries can be directed to the corresponding author.
