# Supplementary material for: Computational insights into the inhibitory mechanism of type 2 diabetes mellitus by bioactive components of Oryza sativa L. indica (black rice)
Source: Front Pharmacol. 2024 Sep 23;15:1457383. doi: 10.3389/fphar.2024.1457383 (PMC11459461; doi:10.3389/fphar.2024.1457383)

**Supplementary Figure 1**

GC-MS results of three compounds that were shortlisted for simulations

C14


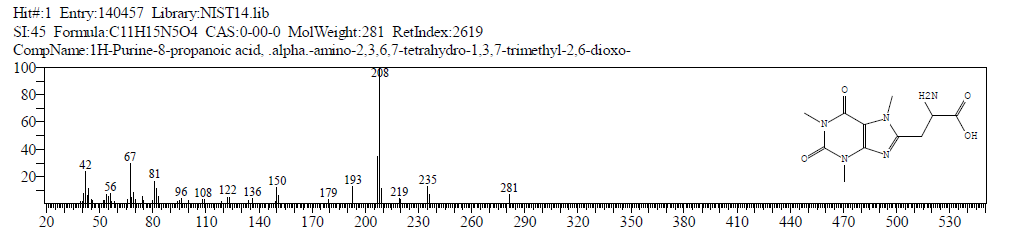


C15


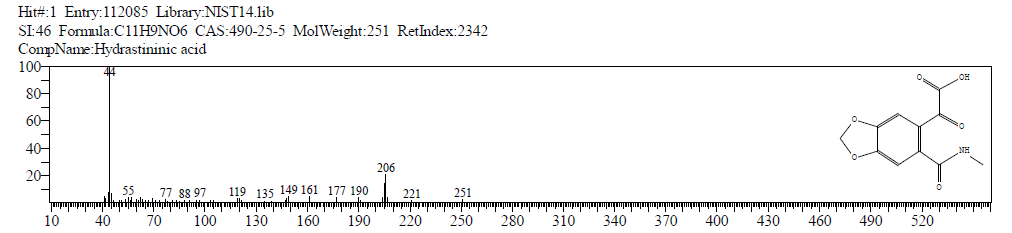


C18


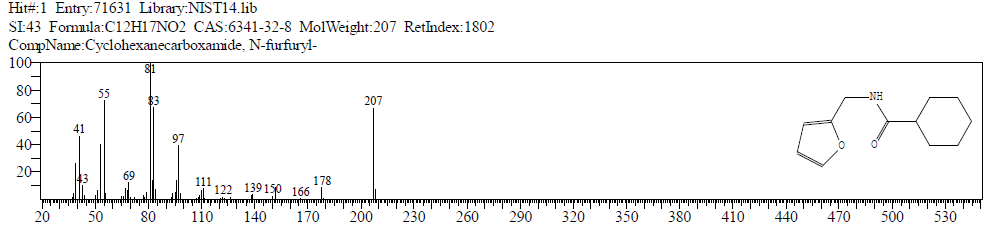

Supplement: Supplementary file 1 [file Table1.DOCX]
